# Oral administration of *Clostridium butyricum* rescues streptomycin-exacerbated respiratory syncytial virus-induced lung inflammation in mice

**DOI:** 10.1080/21505594.2021.1962137

**Published:** 2021-08-12

**Authors:** Wenwen Zhu, Jia Wang, Na Zhao, Rui Zheng, Dalu Wang, Weiwei Liu, Beixing Liu

**Affiliations:** Department of Pathogenic Biology, School of Basic Medical Science, China Medical University, Shenyang, China

**Keywords:** Gastrointestinal microbiota, RSV, immune response, macrophages, *Clostridium butyricum*, butyrate

## Abstract

Changes in the intestinal microbiota indirectly impact the health of mucosa distal to the intestine, particularly the respiratory tract. However, the effects of intestinal microbiota dysbiosis on the regulation of respiratory syncytial virus (RSV) infection are not clear. In this study, we examined the effects of altering the intestinal microbiota on the pulmonary immune response against RSV infection. BALB/c mice were treated with streptomycin before infection with RSV to study the altered immune response. The ingestion of streptomycin led to a marked alteration in the intestinal microbiota with a reduced abundance of *Lactobacillus* and *Clostridium* genera, followed by greatly aggravated pulmonary inflammation in response to RSV infection. This aggravated inflammation was associated with a dysregulated immune response against RSV infection, characterized by the increased expression of IFN-γ and IL-17 and increased pulmonary M1-like macrophage polarization, and decreased expression of IL-5. Supplementation of *Clostridium butyricum* (CB) prevented aggravated inflammation and the dysregulated immune response characterized by greater M2 polarization of pulmonary macrophages and decreased release of IFN-γ and IL-17 as well as increased IL-5 levels. Furthermore, *in vitro* and *in vivo* experiments identified that butyrate, the main metabolite produced by CB, promoted M2 polarization of macrophages in RSV-infected mice exposed to streptomycin. Together, these results demonstrate the mechanism by which intestinal microbiota modulate the pulmonary immune response to RSV infection.

## Introduction

Respiratory syncytial virus (RSV), a negative-sense and single-stranded paramyxovirus virus with a filamentous envelope belonging to the Orthopneumovirus genus of family Pneumoviridae, is a widespread pathogen, that infects almost all children by age 2, with half of the children experiencing two infections during that time [1]. Severe RSV infection can affect airway function through viral and inflammatory damage [2,3], resulting in asthma-like inflammation, such as airway hyper-responsiveness (AHR), inflammatory cell infiltration, especially eosinophils, and pro-inflammatory cytokine production [4]. Although the exact mechanism of RSV-induced pathogenesis is not clear, the relative superiority of Th2 cytokines over Th1 cytokines has been shown to be critical for the occurrence of asthma and exacerbation of asthma caused by RSV infection [5–7].

Recognition of the significant disease burden posed by RSV has led to the need to better understand what drives severe disease in order to develop new effective interventions. Increasing evidence in human and animal models suggests that gut microbiota dysbiosis has a profound influence on distant mucosal site to the intestine, particularly in respiratory tract health. Clinical trials have shown that supplementation of probiotics alters the gut microbiome [8,9] and reduces the incidence of rhinovirus infection in premature infants as well as the duration and severity of rhinovirus infection in adult [10]. In mice, by using germ-free mice [11,12] or antibiotics [13], the effects of changes in the gut microbiota on asthma susceptibility were well established [13–16]. Moreover, gut microbiota dysbiosis induced by antibiotic treatment inhibits lung interferons and T cell immunity, leading to immune impairment and increased pulmonary viral load after influenza virus [17,18]. Similar results were seen in Sendai virus (SeV) infection, in which antibiotic treatment led to a marked dysbiosis in gut microbiome and significantly induced a dysregulated immune response characterized by decreased lung and intestinal regulatory T cells and increased lung interferon (IFN)-γ, resulting in an increase in mortality of SeV-infected mice [19]. Therefore, it is necessary to clarify the connection between gut microbiota and RSV infection.

Several reports have studied the association of gut microbiota with RSV infection. Groves and his colleagues revealed that RSV infection altered the gut microbiota, providing a favorable environment for the S24_7 family, suggesting a link between the S24_7 family and RSV infection [20]. The gut microbiota of patients with moderate and severe RSV are enriched wtih S24_7, Clostridiales, Odoribacteracae, and Lactobacillaceae relative to healthy individuals [21]. However, no studies have addressed the role of the gut microbiota on the pulmonary immune response to RSV infection. Herein, we attempted to determine whether antibiotic perturbation of the gut microbiota would lead to changes in the respiratory immune response against RSV infection in murine models.

## Methods

### Mice and RSV inoculation

Six-week-old female BALB/c mice (Shanghai Laboratory Animal Center, Shanghai, China) were bred and maintained in a specific-pathogen-free facility at the Laboratory Animal Center, China Medical University. All experiments were implemented in accordance with the guidelines and regulations approved by the Institutional Animal Care and Use Committee of China Medical University, China. The human RSV type A2 (RSV A2) strain was propagated in HEP-2 cells (ATCC). Virus titer was expressed as a 50% tissue culture infection dose (TCID50), calculated using the method of Reed and Muench [22]. For RSV inoculation, mice were inoculated intranasally at an inoculum dose of 2 × 10^6^ TCID50 per mouse in 20 μL of sterile phosphate-buffered saline (PBS). As controls, mice were inoculated intranasally with sterile PBS.

### Antibiotic and probiotic treatment

BALB/c mice were given either reverse osmosis water (RO water) or RO water supplemented with 0.5 g streptomycin sulfate (MACKLIN) per 250 mL fresh water for 2 weeks. Water bottles containing streptomycin were replaced twice weekly. *Clostridium butyricum* (BNCC, 337,239; 200 μL/day, concentration of live bacteria, 1 × 10^8^ CFU/mL) was continuously administered via the intragastric route for 1 week before RSV infection. Sodium butyrate (Sigma, 303,410; 200 μL/day, concentration of 3 M) was continuously administered via intragastric route for 1 week before RSV infection. The mice were euthanized on day 3 after RSV infection, and the samples were collected.

### Bronchoalveolar lavage and histopathology

Bronchoalveolar lavage (BAL) was performed on day 3 after RSV infection. The lungs were lavaged two times with 1 mL PBS to collect BAL fluid (BALF). BALF was centrifuged at 2000 rpm for 5 min at 4°C. The cell precipitate was resuspended with 100 PBS and 10 μL was taken for counting the total number of inflammatory cells in BALF. The remaining cell suspension was smeared at 1000 rpm for 5 min at 4°C with a cell centrifuge smear (Thermo Shandon Cytospin 4) and then stained with Wright Giemsa stain. A total of 200 cells per slide were counted, and the proportions of different leucocytes were calculated. Whole lungs were then removed and fixed in 10% formalin, embedded in paraffin. The lung tissue sections (5-μm) were stained with hematoxylin and eosin (H&E) for analysis of peribronchial cellular infiltration.

### Lung single cell preparation

After anesthesia, mice were flushed the lung circulation with 20 mL of sterile PBS to remove intravascular blood pool. Removed lungs were minced and incubated at 37°C on a rocker for 60 minutes with 200 mg/mL collagenase D and 40 mg/mL DNase I (Roche). Then, the enzyme-digested lung tissue was passed through stainless steel mesh. Lung single-cell suspensions were collected by density-gradient centrifugation with lymphocyte-separation solution.

### Cell culture and treatment

The murine macrophage cell line, RAW 264.7 was cultured in Dulbecco’s modified Eagle’s medium (DMEM) with 10% FBS and infected with RSV at multiplicity of infection (MOI) of 2 for 1 h. RAW 264.7 cells were pre-treated with or without butyrate (0.3, 1, or 3 mM). The mRNA expression was detected by real-time PCR at 48 h after RSV infection.

### RNA isolation and real-time PCR

Total RNA was extracted from lung homogenate or purified cells with TRIzol reagent (Life Technologies) and was converted to cDNA using SuperScript III Reverse Transcriptase (Life Technologies). Quantitative real-time PCR was performed using SYBR Green Master Mix (Life Technologies). Primer sets for individual genes are shown in Supplementary Table 1. Real-time PCR was run in a LightCycler® 480 (Roche Molecular Biochemicals) under identical amplification conditions. The results were normalized to β-actin expression and presented as fold change (fold change = 2^−∆∆CT^).

### Flow cytometry and magnetic separation

Lung single-cell suspensions were blocked with anti-mouse CD16/32 (BD Bioscience) and then, stained with fluorochrome-conjugated antibodies against CD45, F4/80, CD11b, CD86, CD206, and ST2 (BD Bioscience). Lineage cocktail included antibodies to CD3, CD4, CD5, CD8, CD11b, Gr-1, CD19, B220, DX5, and TCRδ (BD Bioscience). To obtain lung macrophages, single-cell suspensions from the lungs of tested mice were separated by magnetic bead purification of F4/80 positive cells (Invitrogen, Thermo Fisher Scientific, CA 8802–6863).

### Microbial analysis

For composition analysis, feces from the cecum of mice were removed, and total DNA was extracted using E.Z.N.A. ® Soil DNA Kit (OMEGA) following the manufacturer’s protocol. 16S rRNA gene fragments were amplified using the universal primer sequence 341 F (5ʹ-CCTACGGGNGGCWGCAG-3ʹ) and 805 R (5ʹ-GACTACHVGGGTATCTAATCC-3ʹ). The PCR products were purified using an Agencourt AMPure XP Beads Kit (Beckman Coulter Inc., Brea, CA, USA). The resulting 16S rRNA gene sequences were selected to compare the relative abundance of bacterial taxa. Global community structure comparisons from feces samples were made using PCoA and Simpson’s diversity index implemented using mothur (version 1.30.1) [23]. Taxonomy was assigned using the RDP Classifier (version 2.12) at a confidence threshold of 80% [24]. The differential abundance analysis of taxa was performed at genus levels by Welch’s t-test using STAMP software (version 2.1.3) [25]. The raw sequence data reported in this paper have been uploaded to the NCBI large capacity database SRA (Sequence Read Archive, https://www.ncbi.nlm.nih.gov/sra/PRJNA669372 Accession: PRJNA669372).

### Statistical analysis

Differences between control and experimental groups were compared using one-way analysis of variance (ANOVA) to calculate the statistical significance (GraphPad Prism software, version 7.0).

## Results

### Alteration of gut microbiota caused by streptomycin

**Figure 1.**
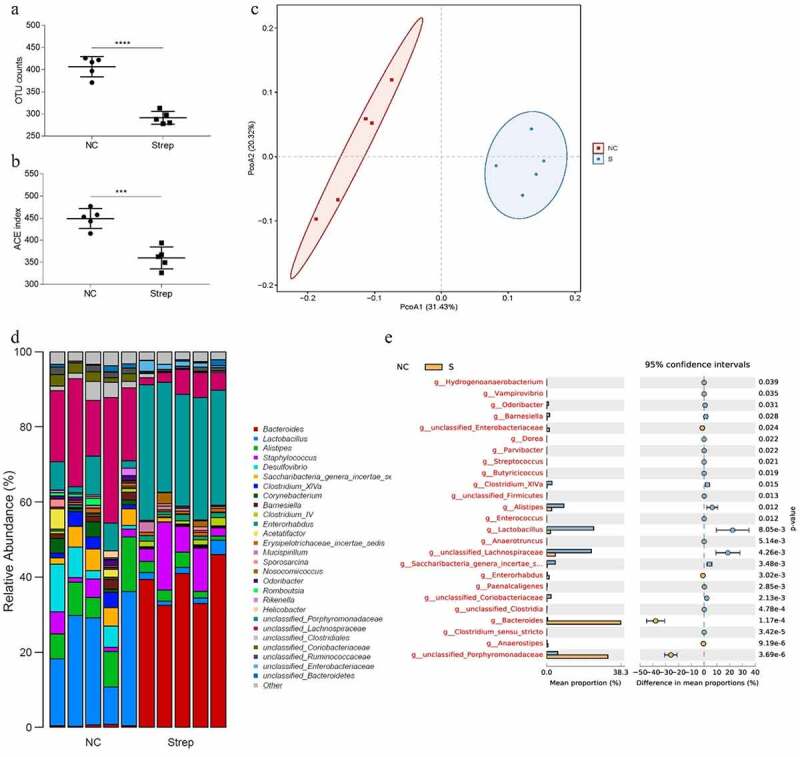
Antibiotic treatment alters intestinal bacterial communities. Operational taxonomic unit (OTU) counts (a), Alpha diversity (b) and Beta diversity (c) using principal coordinate analysis (PCoA) were compared between the controls (NC) and streptomycin-treated groups. Relative abundance of intestinal bacteria taxa at the genus level (d). Differential abundance of bacteria taxa based on genera in fecal microbiota of NC and streptomycin-treated mice (e)

Changes in the gut microbiota composition after streptomycin treatment were assessed by 16S high-throughput sequencing, which revealed significantly different profiles between streptomycin-treated mice and control animals. Alpha diversity and beta diversity showed significant differences in microbiota diversity and composition among the two groups (Figure 1(a–c)). Moreover, streptomycin-treated and control mice exhibited different microbiota profiles. Interestingly, we noticed a significant decrease in the relative abundance of the genera *Lactobacillus, Clostridium_XlVa, Alistipes*, and enriched *Bacteroides* following streptomycin treatment (Figure 1(d,e)).

### Streptomycin treatment aggravates RSV-induced lung inflammation

**Figure 2.**
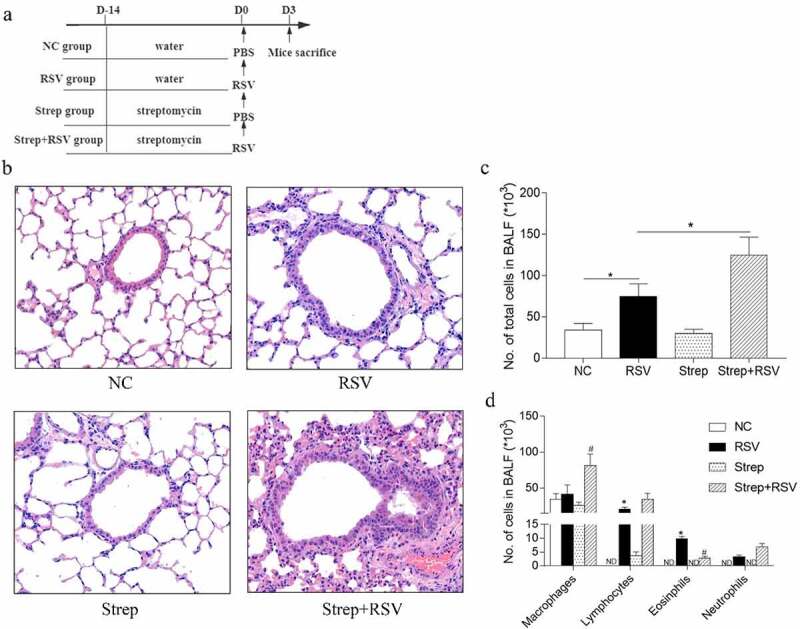
Streptomycin treatment results in enhanced pulmonary peribronchial inflammation following RSV infection. BALB/c mice were placed on a RO or RO+streptomycin water diets, and 2 weeks later were infected intranasally with RSV. Experimental design (a). Images of H&E-stained lung sections showing peribronchial inflammation (b). Total (c) and different leukocytes subtypes (d) in BAL fluids. Data are presented as the mean ± SD of results for each group of 3 mice tested. *p < 0.05 by ANOVA was considered statistically significant. ND, not detectable

RSV infection induces pulmonary inflammatory cell infiltration and pulmonary virus proliferation. To determine whether antibiotic perturbation of intestinal microbiota would affect RSV infection, mice were infected with RSV mice after exposure to streptomycin for 2 weeks (Figure 2(a)). Analysis of BAL and lung tissue pathology was then performed. Consistent with our previous finding in RSV-infected mice [26], RSV induced a significant increase in the number of total inflammatory cells, which was due to the increase of eosinophils, lymphocytes, and neutrophils compared with normal controls (Figure 2(c,d)). However, RSV-challenged mice treated with streptomycin resulted in higher total inflammatory cells in BAL compared to RSV-challenged control mice (Figure 2(c)). Differential type analysis of the infiltrating inflammatory cells in BAL revealed that streptomycin-treated RSV-infected mice had increased macrophages and neutrophils as well as decreased eosinophils relative to controls (Figure 2(d)). As expected, no difference in total inflammatory cells or cell type was observed in normal control or streptomycin-treated mice. Alveolar macrophages made up the majority of cells in the BAL.

The preliminary analysis of lung inflammation in the model mice based on BAL results was further confirmed by pulmonary histopathology. RSV-induced inflammatory cell infiltration was predominantly localized to the bronchus, whereas streptomycin-treated RSV-challenged mice showed severe inflammation in the lungs (Figure 2(b)). No pathological changes were observed in normal control mice or in streptomycin-treated control mice. Moreover, streptomycin treatment had no effect on viral load in the lungs of mice (Supplementary Figure 1). Taken together, streptomycin treatment could enhance the severity of inflammation in the lungs of RSV-infected mice.

### Streptomycin treatment led to a dysregulated lung immune response to RSV

**Figure 3.**
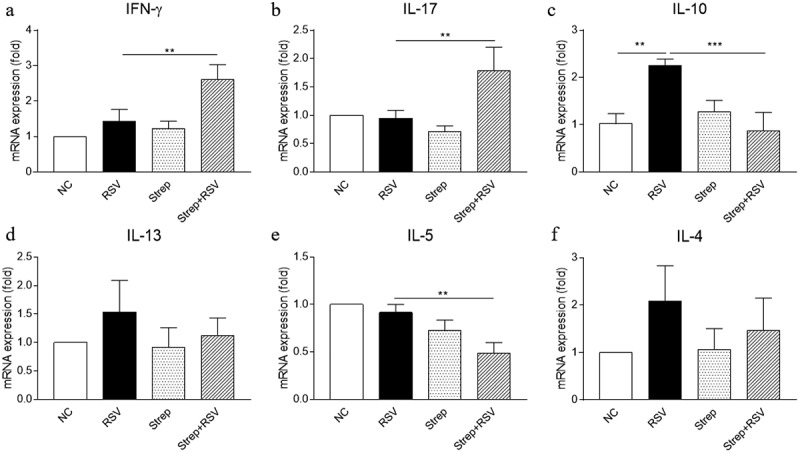
Streptomycin treatment alters pulmonary cytokines response to RSV. BALB/c mice were placed on RO or RO+streptomycin water diet, 2 weeks later were infected with RSV. The relative gene expression of IFN-γ (a), IL-17 (b), IL-10 (c), IL-13 (d), IL-5 (e), and IL-4 (f) in lung tissues were determined by real-time PCR method. Data are reported as mean ± SD of the results for each group. *p < 0.05 and **p < 0.01 by ANOVA were considered statistically significant

One possible cause for aggravated inflammation in RSV-infected mice exposed to streptomycin would be the dysregulation of the immune response. It is well established that RSV infection induces predominantly Th2-associated pulmonary inflammation, such as the production of IL-13, IL-4, and IL-5 [27] [28,29]. Interestingly, we found significantly increased mRNA expression of IFN-γ and IL-17A as well as decreased expression of IL-10 in the lungs of streptomycin-treated RSV-challenged mice relative to RO-treated RSV-challenged mice (Figure 3(a,b)). Lung tissue from streptomycin-treated mice tended to secrete less IL-13, IL-4, and IL-5 after RSV infection (Figure 3); however, these differences were not statistically significant, except for the differences in IL-5 expression (*P* < 0.01) (Figure 3(e)). There were no differences in the production of these inflammatory cytokines in streptomycin-treated mice compared with normal control animals, which suggested that the effect of streptomycin treatment on RSV-infected mice was not caused by the direct effect of streptomycin on lung tissue. In summary, we found that streptomycin treatment influenced RSV-induced pulmonary inflammation by skewing Th2 switching toward a Th1- or Th17-associated response.

### Macrophage activation in the lungs likely contributed to streptomycin-exacerbated RSV-induced lung inflammation

**Figure 4.**
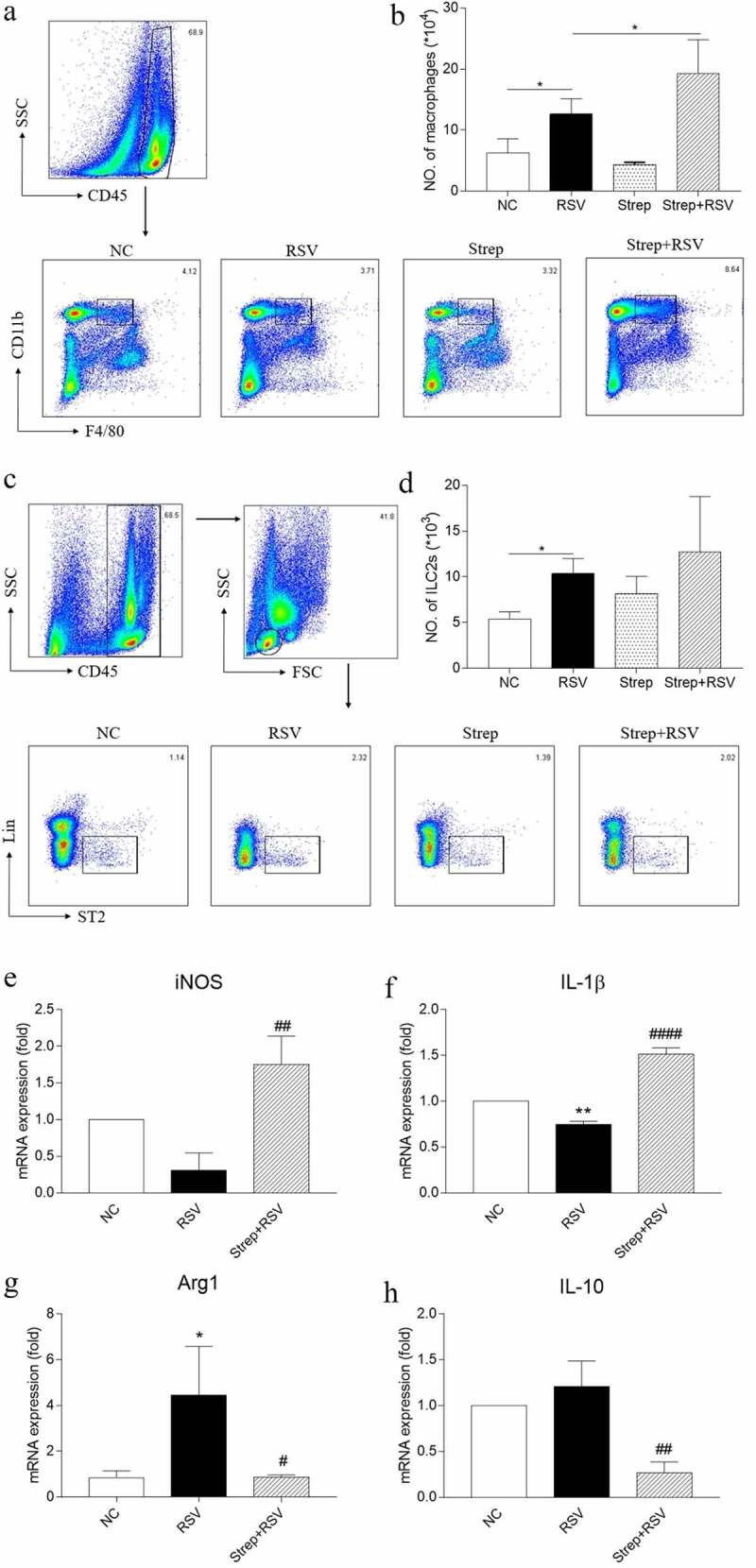
Streptomycin treatment augments the number of pulmonary macrophages and the expression of M1 cytokines in pulmonary macrophages during RSV infection. On day 3 after infection, lung cells were collected, and pulmonary macrophages were isolated by magnetic bead purification. Gating strategy of pulmonary macrophages (a) and ILC2s (c). Total number of pulmonary macrophages (b) and ILC2s (d) were determined by flow cytometry. Expression of iNOS (e), IL-1β (f), Arg1 (g), and IL-10 (h) mRNA relative to the expression of β-actin in pulmonary macrophages were detected by real-time PCR. Data are reported as mean ± SD of the results for each group of 5 mice tested. *p < 0.05 and **p < 0.01 by ANOVA compared with NC group and #*p* < 0.05, ##*p* < 0.01, ####*p* < 0.0001 by ANOVA, compared with RSV group were considered statistically significant

Several studies have reported the regulating effect of the gut microbiota on innate immunity and its impact on inflammation [30,31]. To determine whether exacerbated RSV-induced lung inflammation in streptomycin-treated mice was due to changes in innate immune responses, we compared innate immune cells in the lungs of streptomycin-treated and RO-treated mice after a 3-d exposure to RSV. As Group 2 innate lymphoid cells (ILC2s) have been implicated in RSV infection [32,33], we investigated the prevalence of these lymphocytes in the lung of streptomycin-treated or RO-treated RSV-challenged mice. ILC2s showed a significant increase after RSV infection in RO-treated mice (Figure 4(c,d)); however, no differences were observed in streptomycin-treated RSV-challenged mice compared with RO-treated RSV-challenged animals (Figure 4(c,d)). In an attempt to determine whether other innate immune cells took part in streptomycin-induced aggravation of RSV inflammation, we also compared the levels of CD45^+^F4/80^+^CD11b^+^ macrophages, which play a critical role in the modulation of pulmonary inflammation during respiratory viral infection [34], in the lungs of streptomycin-treated and RO-treated mice. Interestingly, we found macrophages to be strikingly increased in the streptomycin-treated RSV-challenged mice relative to RO-treated RSV-challenged mice (Figure 4(a,b)). Consistent with above results, streptomycin treatment did not cause any changes in the frequency of ILC2s or macrophages in the lungs of normal animals compared with RO-treated mice.

To further confirm the potential role of macrophages, lung macrophages were collected from streptomycin-treated or RO-treated mice at day 3 after RSV infection to test the relative levels of activated cytokine secretion. We found RSV infection could promote lung macrophages to secrete more arginase-1 (Arg1) but less IL-1β and inducible nitric oxide synthase (iNOS). However, streptomycin treatment reversed this situation, showing less expression of Arg1 and IL-10, with more expression of IL-1β and iNOS in the lungs of RSV-infected mice (Figure 4(e–h)), suggesting that M1-polarization of macrophages may be responsible for the exacerbation of lung inflammation of streptomycin-treated RSV-challenged mice.

### *Clostridium butyricum* supplements rescued streptomycin-induced aggravation of inflammation in RSV-infected mice

Given that streptomycin-treated mice presented a more severe RSV infection than control mice, the gut microbiota that were reduced or enriched after streptomycin treatment were considered to influence disease-susceptible bacteria. In the present study, streptomycin treatment reduced *Clostridium* genera, which is one of the most important genera to produce abundant butyrate [35] and has been reported to modulate the functions of extra-intestinal immune cells, such as Tregs [36] and macrophages [37]. Thus, we speculated that the reduction in *Clostridium* spp. in the gut after streptomycin may be the primary cause of changes in RSV-induced immune response in the lung.

**Figure 5.**
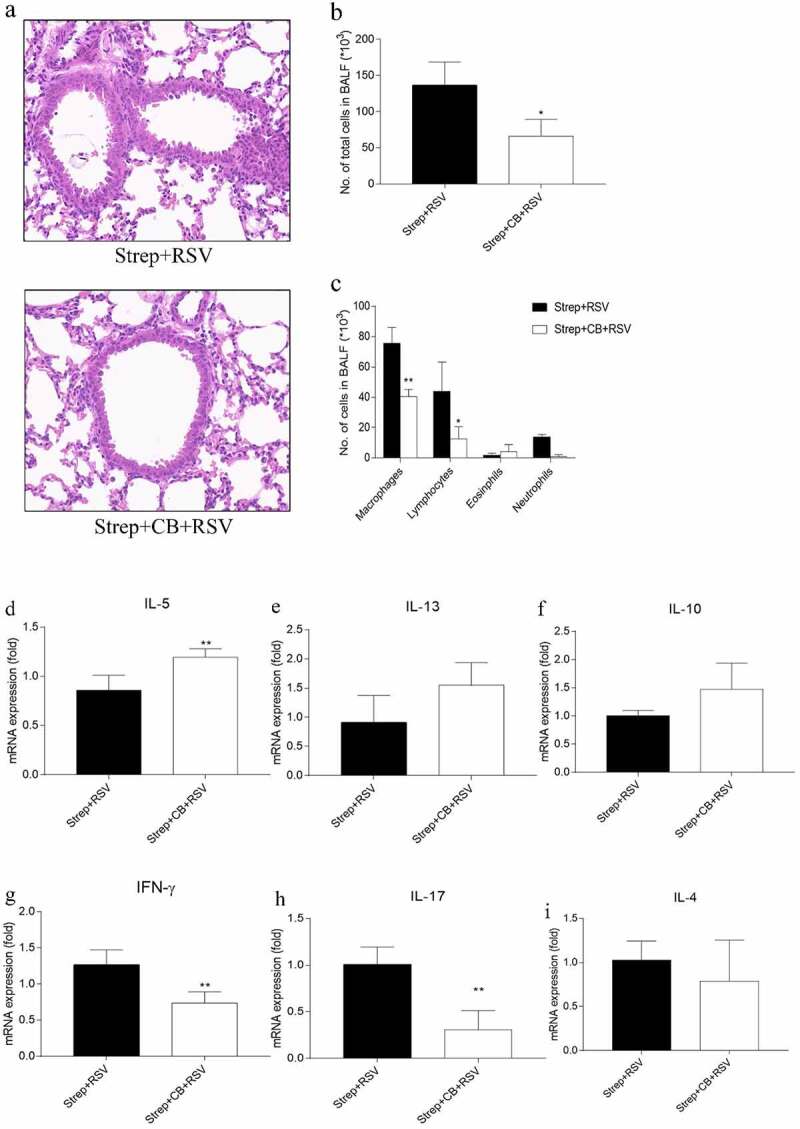
CB supplement reduces pulmonary inflammation (b-d) and alters the pulmonary cytokine response (e-j) to RSV infection in streptomycin-treated mice. Images of H&E-stained lung sections showing peribronchial inflammation (a). The number of total cells (b) and different leukocyte subtypes (c) in BAL fluids. The relative expression of mRNAs for IL-5(d), IL-13 (e, IL-10 (f), IFN-γ (g), IL-17 (h) and IL-4 (i) in lung tissues was determined by real-time PCR. Data are reported as the mean ± SD of the results for each group. *p < 0.05 and **p < 0.01 by ANOVA were considered statistically significant

To provide evidence for our speculation, mice that had received a 2-week streptomycin treatment were intragastrically given *Clostridium butyricum* (CB), which belongs to *Clostridium* genus named for its capacity to produce high amounts of butyric acid in the gut [38], for 1 week, and then infected with RSV. BAL analysis and lung tissue histopathology were performed. Surprisingly, the CB supplement reduced the overall amount of inflammatory cells in the BAL (Figure 5(b)) as well as lung viral load (Supplementary Figure 1) of streptomycin-treated RSV-challenged mice to that of RO-treated RSV-challenged mice. Differential analysis of the infiltrating inflammatory cells in the BAL showed that exposure to CB also reduced the levels of macrophages, lymphocytes, and neutrophils of streptomycin-treated mice (Figure 5c). Moreover, CB also alleviated inflammatory cell infiltration in lungs of streptomycin-treated mice as evidenced by H&E staining (Figure 5a).

### CB restored the inflammatory response type and decreased the number of lung macrophages

We next questioned whether the dysregulated cytokine response in RSV-infected streptomycin-treated mice was restored following exposure to CB supplements. The expression of IL-13, IL-4, IL-5, IFN-γ, IL-17A, and IL-10 in the lungs of tested mice was measured again. As expected, the level of IFN-γ and IL-17A decreased significantly in CB-treated mice relative to controls (Figure 5(g,h)). Lung tissues from CB-treated mice tended to express more Th2 cytokines, with only IL-5 expression showing a significant difference compared with streptomycin-treated animals (Figure 5(d)).

**Figure 6.**
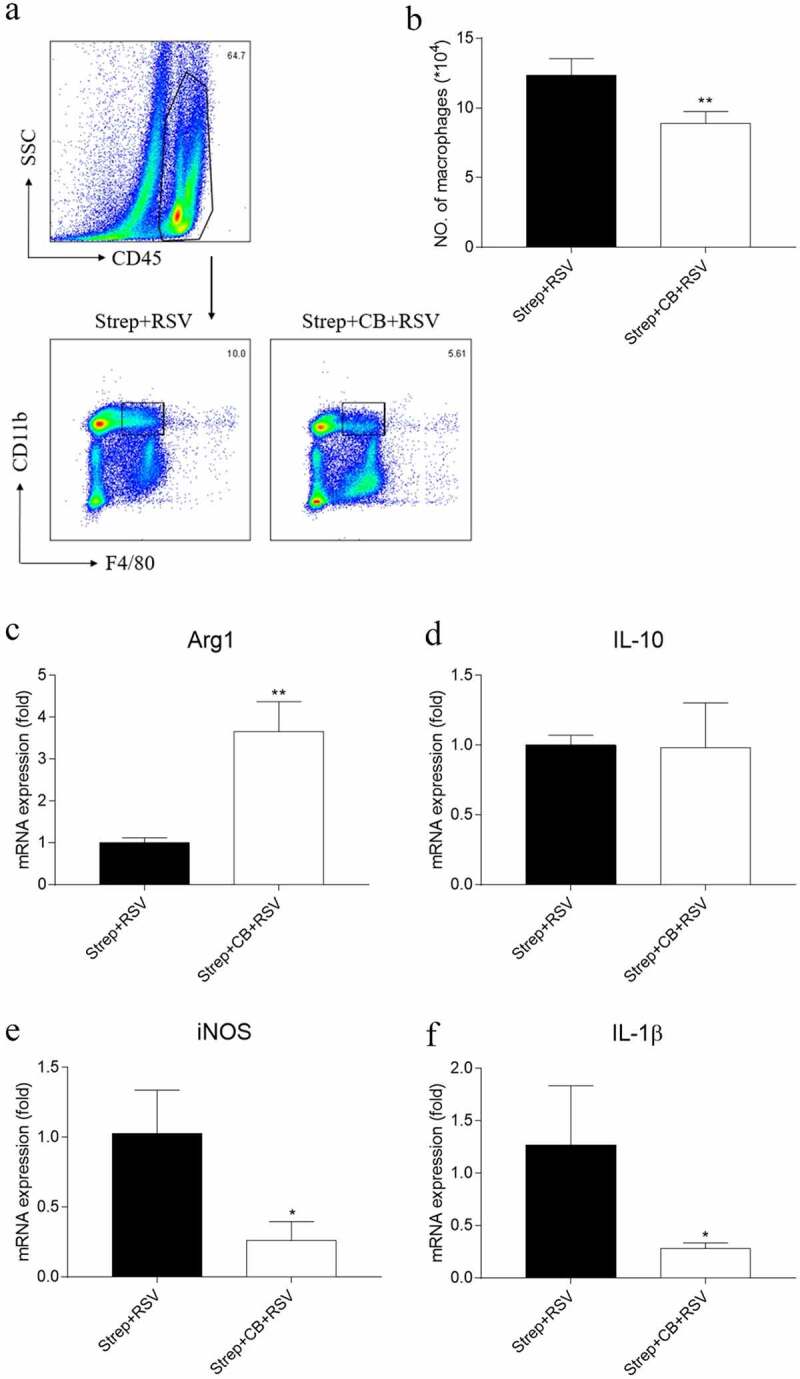
CB supplement reduces the number of pulmonary macrophages and the expression of M1 cytokines in macrophages in response to RSV infection in streptomycin-treated mice. Gating strategy of pulmonary macrophages (a). Total pulmonary macrophages (b) were determined by flow cytometry. The gene expression of Arg1 (c), IL-10 (d), iNOS (e) and IL-1β (f) relative to the expression of β-actin in pulmonary macrophages was detected by real-time PCR. Data are reported as mean ± SD of the results for each group of 5 mice tested. *p < 0.05, **p < 0.01 by ANOVA compared with NC group, were considered statistically significant

We also detected changes in macrophages in the lungs of tested mice. Similarly, CB supplement significantly reduced the prevalence of macrophages compared to streptomycin-treated mice after RSV infection (Figure 6(a,b)). Further evaluation of isolated macrophages revealed that the streptomycin-induced increase in iNOS and IL-1β secretion was significantly reduced after CB supplement, and the reduced mRNA expression of Arg1 was increased significantly (Figure 6(c,e,f)). However, IL-10 expression level did not change after CB supplement (Figure 6(d)).

In summary, CB supplements diminished the effects of streptomycin treatment on the lung of RSV-infected mice, and mimicked the pulmonary immune response similar to the levels before streptomycin treatment.

### Sodium butyrate regulated the expression of pro- and anti-inflammatory cytokines in RAW 264.7 *in vitro*

**Figure 7.**
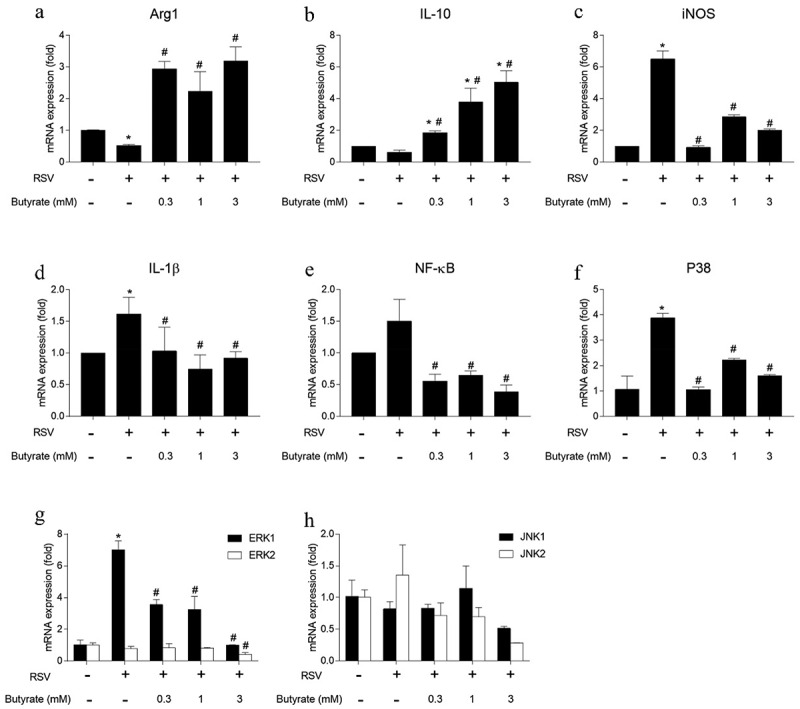
Butyrate modulates RSV-infected murine macrophages. RAW 264.7 cells (5 × 10^5^ cells/mL) were infected with RSV at MOI: 2 in the presence or absence of butyrate for 24 h and further incubates for 48 hours. The expression of Arg1 (a), IL-10 (b), iNOS (c), IL-1β (d), NF-κB (e), P38 (f), ERK1/2 (g), and JNK1/2 (h) mRNA were respectively determined by real-time PCR. *p < 0.05 by ANOVA, compared with the control (NC) group and #*p* < 0.05 by ANOVA compared with RSV group without butyrate were considered statistically significant

To clarify the potential mechanism involved in CB supplementation changes the polarization of macrophages in the lungs of RSV-infected mice, we evaluated the role of butyrate on RSV-infected RAW264.7 murine macrophages as the main metabolite of CB in the intestinal tract is butyrate. RAW264.7 cells were stimulated with RSV in the presence or absence of butyrate for 24 h and were then further incubated for 48 h to examine the ability of butyrate to regulate RSV-induced M1- or M2-associated marker production in macrophages. The infection of RAW264.7 cells with RSV led to an increased expression of iNOS and IL-1β, as well as the decreased expression of Arg-1 (Figure 7). Butyrate remarkably inhibited RSV-induced iNOS and IL-1β mRNA expression levels but promoted Arg-1 and IL-10 production in RAW264.7 cells (Figure 7). These results indicated that butyrate could downregulate RSV-induced iNOS and IL-1β levels but upregulated Arg-1 and IL-10 expression in murine macrophages.

Previously, we found that RSV infection activated the MAPK signal pathways p38, JNK1/2, and ERK1/2 in RAW264.7 cells [39]. In addition, NF-κB activation is an essential signaling pathway responsible for iNOS expression [40]. We therefore sought to determine whether the expression of mRNAs for these signal molecules changed. The expression of ERK1 and p38 mRNA in RAW264.7 increased following RSV infection (Figure 7). Butyrate significantly inhibited the expression of NF-κB, p38, and ERK1, especially at the 3 mM treatment. JNK1/2, ERK2 did not show any further changes. Altogether, butyrate may regulate the expression of pro- and anti-inflammatory cytokines in RSV-stimulated RAW264.7 cells by inhibiting the NF-κB, p38, and ERK1 signaling pathways.

### Sodium butyrate supplementation promoted M2 polarization of pulmonary macrophages in vivo

**Figure 8.**
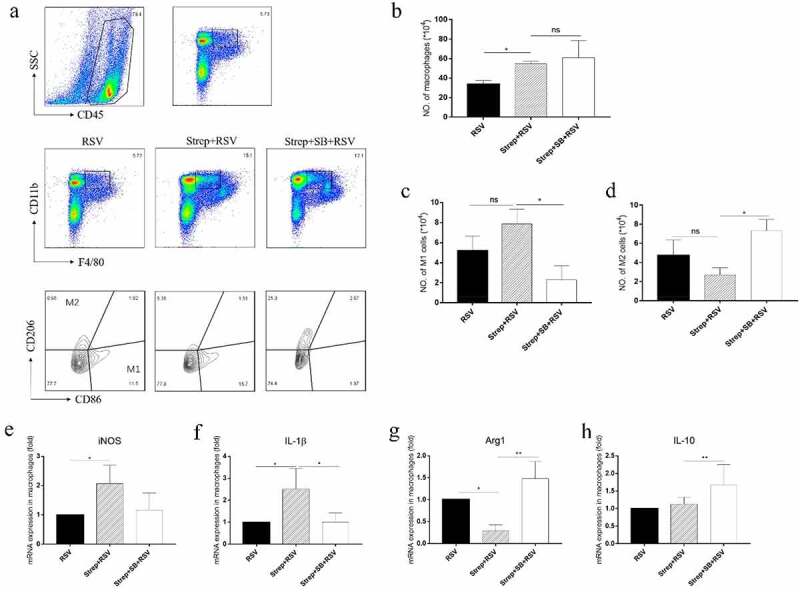
Sodium butyrate (SB) supplement promotes M2 polarization in response to RSV infection in streptomycin-treated mice. Gating strategy of pulmonary macrophages and M1/M2 macrophages (a). Total pulmonary macrophages (b) and M1/M2 type macrophages (c, d) were determined by flow cytometry. The gene expression of iNOS (e), IL-1β (f), Arg1 (g) and IL-10 (h) relative to the expression of β-actin in pulmonary macrophages was detected by real-time PCR. Data are reported as mean ± SD of the results for each group of 5 mice tested. *p < 0.05, **p < 0.01 by ANOVA were considered statistically significant

To further clarify the role of butyrate in regulating macrophage activation in vivo, mice that had received a 2-week streptomycin treatment were intragastrically given sodium butyrate (SB) for 1 week, and then infected with RSV. Pulmonary macrophages were analyzed by flow cytometry. Results showed that SB supplementation did not significantly affect the amount of pulmonary macrophages, but alters the polarization of macrophages in streptomycin-treated RSV-infected mice (Figure 8(a–d)). Further evaluation of isolated pulmonary macrophages revealed that the streptomycin-induced increased in IL-1β secretion was significantly reduced after SB supplement, and the mRNA expression of Arg1 and IL-10 was increased significantly (Figure 8(f–h)). These results indicated the efficacy of SB in modulating pulmonary macrophage polarization from M1 to M2 in streptomycin-exacerbated RSV infection.

## Discussion

In the past few decades, the importance and universality of the gut microbiota have been recognized, and much effort has focused on identifying the bacterial populations associated with the development of respiratory disease, especially respiratory viral infections. The findings from different studies indicated that the mechanisms regulating the influence of the gut microbiota on respiratory viral infection differed significantly likely due to the differences in the experimental models and methods. Nevertheless, most studies pointed toward the notion that an intact gut microbiota is conductive to the host’s antiviral immunity [18,19]. In the present study, we propose a novel role for the lung-gut axis using an RSV-infected mice model. Our study used a more classical approach: by creating detectable changes in the gut microbiota exposed to antibiotic before RSV infection, we identified members of the microbiota that may disturb RSV-induced pulmonary inflammation and the underlying mechanisms.

Classically, RSV infection induces a Th2-driven immune response, mainly represented by the production of Th2 cytokines (IL-4, IL-5, IL-10, and IL-13) and the recruitment of eosinophils in lung tissue, which correlated with the generation of M2 macrophages during RSV infection [41,42]. This is consistent with our results which showed increased eosinophils in BALF and increased expression of IL-4, IL-13, and IL-10 in the lung tissue, as well as enhanced expression of Arg1 in isolated pulmonary macrophages indicative of M2 polarization in RSV-infected mice. However, in this study, mice pretreated with streptomycin produced a different pulmonary immune reaction in response to experimental RSV infection. The severity of RSV infection was exacerbated by streptomycin treatment as evidenced by more severe pulmonary histopathology and increased infiltration of inflammatory cells and macrophages in BAL, which reflected the degree of airway inflammation, and was accompanied by enhanced expression of IFN-γ and IL-17A (IL-17). Correspondingly, increased presence of macrophages and greater M1 polarization in the lung tissue were also found. Our results suggested that streptomycin treatment skewed the immune response toward Th1 and Th17 pathways and promoted pulmonary macrophages toward an M1 phenotype in the lungs of RSV-infected mice, which is consistent with previous study that streptomycin treatment significantly increased pulmonary IFN-γ production and reduced regulatory cells, resulting in an increase of mortality in SeV-infected mice [19]. In addition, the pleiotropic functions of IFN-γ in inducing a robust Th1 response and differentiation of M1-like macrophages have been revealed [43–45]. M1 macrophages play a central role in the cytotoxicity of microorganisms and induce elevated production of pro-inflammatory cytokines such as iNOS and IL-1β, as well as skew the immune response to Th1 [42,46]. In contrast, M2 macrophages represent potent effector cells involved in Th2 response or regulatory IL-10-associated immunity, thereby inhibiting the inflammatory response, and promoting tissue modeling [47,48]. Moreover, as previously mentioned, IL-17 is an inflammatory cytokine that drives neutrophil recruitment and activation, and in addition, it is correlated with severe RSV infection and exacerbation of allergic disease [49,50]. IL-17 levels mediate inflammation and airway remolding and are associated with asthma health outcomes [51,52]. Thus, changes in the immune response to a pulmonary viral infection induced by streptomycin treatment revealed a complicate regulatory network that together led to increased pulmonary inflammation.

As an unabsorbable antibiotic, streptomycin has been shown to affect the composition of intestinal microbiota, with only 0.00001% being absorbed when given at a high dosage [53]. Therefore, streptomycin treatment should have little effect on the pulmonary microbiota directly, which strongly supports our results that the pulmonary immune response could be regulated by a disruption of the composition of the intestinal microbiota, which is considered to reflect the lung-gut axis. 16S sequencing of stool samples from mice revealed that oral administration with streptomycin induced broad changes to the composition of intestinal commensal microbiota, which mainly consisted of the reduction of *Firmicutes* phylum (*Lactobacillus, Clostridium_XlVa, Alistipes)* and enrichment of the *Bacteroidetes phylum. Bacteroidetes* is a diverse phylum with limited description of its specific branches. *Bacteroides fragilis* is the most well-studied species in the *Bacteroidetes* phylum, and has been demonstrated to play an important role in the maturation of Th1 immune responses by activating CD4^+^T cells to produce more IFN-γ [54]. Moreover, an enterotoxigenic *B. fragilis* strain promoted colon tumorigenesis in a Th17-dependent manner, further supporting its role in affecting Th17 disease [55].

Although the precise immune mechanism through which streptomycin treatment aggravates RSV-induced inflammation is still unclear, there is strong evidence that members of the intestinal microbiota mediate different immune cell responses, including Treg cells and Th17, and can alter the pathogenesis of variety disease models such as colitis and arthritis [36,56–58]. This has fueled our speculation that changes in the composition of bacterial species may promote the formation of an inflammatory environment that exacerbates the Th1/Th17 response directly or indirectly.

The decreased abundance of the *Clostridium* bacteria caused by streptomycin treatment was confirmed by our study and other studies [59]. Intriguingly, *Clostridium* has been reported to promote the expansion and differentiation of Treg cells *in vitro* and oral administration of a combination of strains falling within clusters IV, XIVa, and XVIII of *Clostridia* in adult mice alleviated disease in models of colitis and allergic diarrhea [36]. Furthermore, another study reported a negative correlation between macrophages and fecal *Clostridium* composition. These data highlight the connection between the reduction of intestinal *Clostridium* and increased macrophages.

Does the decreased abundance of *Clostridium* play a role in the streptomycin-exacerbated RSV inflammation? To test this possibility, streptomycin treated-mice were pretreated with CB, a widespread species belonging to the genus *Clostridium*, prior to RSV infection. We found oral administration of CB supplements was effective in reducing the streptomycin-exacerbated RSV-induced lung inflammation, as evidenced by the significant decrease in the overall levels of inflammatory cells in the BALF, and further alleviated pulmonary histopathology. CB can also significantly suppress the expression of IFN-γ and IL-17 in lung tissue, but also promotes Th2 cytokine expression and induces greater M2 polarization, which may be responsible for the reduced inflammatory response mentioned above. The anti-inflammatory activity of CB has been reported to be involved in intestinal anaphylaxis induced by food allergies [59], and significantly inhibits the allergic inflammation associated with foods in the intestine and contributes to restore the intestinal epithelial barrier functions [60]. Moreover, the efficacy of CB for the treatment of allergic asthma has also been observed in a previous study, which indicated that lung resistance and airway inflammation could be reduced by CB in the asthmatic mice, in addition to the suppression of OVA-specific IgE/G1 expression and mast cell degranulation [61]. Similarly, oral administration of CB supplements ameliorated the gut microbiota dysbiosis mice following RSV infection and was thereby effective in reducing pulmonary inflammation by interfering with the polarization of macrophages to M2 and suppressing pro-inflammatory cytokines in our study.

As the main metabolite produced by CB in the gut, butyrate has been considered the main effector in mediating its anti-inflammatory effects [38,62]. The anti-inflammatory role of butyrate via promoting M2 polarization has been elaborated in many diseases, such as alcoholic liver disease [63], inflammatory bowel disease [64]. Indeed, we also found butyrate could enhance M2 polarization while suppress M1 polarization of macrophages both *in vivo* and *in vitro*. Notably, further mechanistic study revealed that butyrate inhibited the expression of NF-κB, p38, and ERK1 in RSV-infected murine macrophages, indicating the involvement of NF-κB, p38, and ERK1 signaling pathway in its activity. Previous studies have shown that NF-κB and ERK play a crucial role in the regulation of inflammation. They are involved in the modulation of iNOS and the expression of various cytokines in macrophages [65,66]. Additionally, others have also reported that butyrate may inhibit NF-κB and ERK signaling pathways to play anti-inflammatory effects in IFN-γ-stimulated RAW 264.7 cells or LPS-induced RAW 264.7 cells by inhibiting NF-κB and ERK signaling pathways [67,68]. Therefore, it is likely that NF-κB, p38, and ERK1 are target molecules of butyrate in the regulation of RSV-induced pro- and anti-inflammatory cytokine production in macrophages.

In conclusion, this is the first report to describe that antibiotic disruption of intestinal microbiota aggravates pulmonary inflammation in mice infected with RSV via expansion of M1-like macrophages and increased production of pro-inflammatory cytokines. Additionally, supplementation of CB can alleviate pulmonary inflammation by promoting M2 polarization and anti-inflammatory cytokine expression and by inhibiting the expression of pro-inflammatory cytokines. These effects are possibly due to the fact that butyrate, which was produced by CB, is absorbed into bloodstream and inhibits NF-κB, p38, and ERK1 signaling pathways in pulmonary macrophages. These data emphasize the link between the intestinal microbiota and the pulmonary immune system, but they also raise concerns about the potential risks of overuse of antibiotics. Our findings will provide new insight into the development of probiotic treatment in ameliorating RSV infection.

## Supplementary Material

Supplemental Material

## Data Availability

The raw sequence data reported in this paper have been uploaded to the NCBI large capacity database SRA (Sequence Read Archive, https://www.ncbi.nlm.nih.gov/sra/PRJNA669372, Accession: PRJNA669372).

## References

[1] Wp G, Lh T, Al F, et al. Risk of primary infection and reinfection with respiratory syncytial virus. Am J Dis Child. 1986;140(6):543–546. 3706232 10.1001/archpedi.1986.02140200053026

[2] Corne JM, Holgate ST. Mechanisms of virus induced exacerbations of asthma. Thorax. 1997;52(4):380–389. 9196525 10.1136/thx.52.4.380 PMC1758527

[3] Rooney JC, Williams HE. The relationship between proved viral bronchiolitis and subsequent wheezing. J Pediatr. 1971;79(5):744–747. 5116699 10.1016/s0022-3476(71)80385-2

[4] Carroll KN, Gebretsadik T, Escobar GJ, et al. Respiratory syncytial virus immunoprophylaxis in high-risk infants and development of childhood asthma. J Allergy Clin Immunol. 2017;139(1):66–71 e3. 27212083 10.1016/j.jaci.2016.01.055 PMC5074917

[5] Bueno SM, Gonzalez PA, Pacheco R, et al. Host immunity during RSV pathogenesis. Int Immunopharmacol. 2008;8(10):1320–1329. 18687294 10.1016/j.intimp.2008.03.012

[6] Thurau AM, Streckert HJ, Rieger CH, et al. Increased number of T cells committed to IL-5 production after respiratory syncytial virus (RSV) infection of human mononuclear cells in vitro. Clin Exp Immunol. 1998;113(3):450–455. 9737676 10.1046/j.1365-2249.1998.00683.x PMC1905058

[7] Russell CD, Unger SA, Walton M, et al. The human immune response to respiratory syncytial virus infection. Clin Microbiol Rev. 2017;30(2):481–502. 28179378 10.1128/CMR.00090-16 PMC5355638

[8] Khalesi S, Bellissimo N, Vandelanotte C, et al. A review of probiotic supplementation in healthy adults: helpful or hype? Eur J Clin Nutr. 2019;73(1):24–37. 29581563 10.1038/s41430-018-0135-9

[9] de Vrese M, Winkler P, Rautenberg P, et al. Effect of Lactobacillus gasseri PA 16/8, Bifidobacterium longum SP 07/3, B. bifidum MF 20/5 on common cold episodes: a double blind, randomized, controlled trial. Clin Nutr. 2005;24(4):481–491. 16054520 10.1016/j.clnu.2005.02.006

[10] Luoto R, Ruuskanen O, Waris M, et al. Prebiotic and probiotic supplementation prevents rhinovirus infections in preterm infants: a randomized, placebo-controlled trial. J Allergy Clin Immunol. 2014;133(2):405–413. 24131826 10.1016/j.jaci.2013.08.020 PMC7112326

[11] Herbst T, Sichelstiel A, Schar C, et al. Dysregulation of allergic airway inflammation in the absence of microbial colonization. Am J Respir Crit Care Med. 2011;184(2):198–205. 21471101 10.1164/rccm.201010-1574OC

[12] Olszak T, An D, Zeissig S, et al. Microbial exposure during early life has persistent effects on natural killer T cell function. Science (New York, NY). 2012;336(6080):489–493. 10.1126/science.1219328 PMC3437652 22442383

[13] SL R, Gold MJ, Hartmann M, et al. Early life antibiotic-driven changes in microbiota enhance susceptibility to allergic asthma. EMBO Rep. 2012;13(5):440–447. 22422004 10.1038/embor.2012.32 PMC3343350

[14] SL R, MJ G, Bp W, et al. Perinatal antibiotic treatment affects murine microbiota, immune responses and allergic asthma. Gut Microbes. 2013;4(2):158–164. 23333861 10.4161/gmic.23567 PMC3595077

[15] SV L. Gut microbiota and allergic disease. Ann Am Thorac Soc. 2016;13(Suppl 1):S51–4. New Insights 27027953 10.1513/AnnalsATS.201507-451MG PMC5015732

[16] BJ M. Influences of the microbiome on the early origins of allergic asthma. Ann Am Thorac Soc. 2013;10(Suppl):S165–9. 24313768 10.1513/AnnalsATS.201305-118AW

[17] Abt MC, Osborne LC, Monticelli LA, et al. Commensal bacteria calibrate the activation threshold of innate antiviral immunity. Immunity. 2012;37(1):158–170. 22705104 10.1016/j.immuni.2012.04.011 PMC3679670

[18] Ichinohe T, Pang IK, Kumamoto Y, et al. Microbiota regulates immune defense against respiratory tract influenza A virus infection. Proc Natl Acad Sci U S A. 2011;108(13):5354–5359. 21402903 10.1073/pnas.1019378108 PMC3069176

[19] MH G, Camarda LE, Hussain SA, et al. Intestinal microbiota disruption reduces regulatory T cells and increases respiratory viral infection mortality through increased IFNγ production. Front Immunol. 2018;9:1587. 30042764 10.3389/fimmu.2018.01587 PMC6048222

[20] Ht G, Cuthbertson L, James P, et al. Respiratory disease following viral lung infection alters the murine gut microbiota. Front Immunol. 2018;9:182. 29483910 10.3389/fimmu.2018.00182 PMC5816042

[21] JN H, Siefker D, Vu L, et al. Altered gut microbiota in infants is associated with respiratory syncytial virus disease severity. BMC Microbiol. 2020;20(1):140. 32487019 10.1186/s12866-020-01816-5 PMC7268675

[22] Liu B, Kimura Y. Respiratory syncytial virus protects against the subsequent development of Japanese cedar pollen-induced allergic responses. J Med Virol. 2007;79(10):1600–1605. 17705182 10.1002/jmv.20944

[23] Pd S, Westcott SL, Ryabin T, et al. Introducing mothur: open-source, platform-independent, community-supported software for describing and comparing microbial communities. Appl Environ Microbiol. 2009;75(23):7537–7541. 19801464 10.1128/AEM.01541-09 PMC2786419

[24] Wang Q, Garrity GM, Tiedje JM, et al. Naive Bayesian classifier for rapid assignment of rRNA sequences into the new bacterial taxonomy. Appl Environ Microbiol. 2007;73(16):5261–5267. 17586664 10.1128/AEM.00062-07 PMC1950982

[25] Dh P, Tyson GW, Hugenholtz P, et al. STAMP: statistical analysis of taxonomic and functional profiles. Bioinformatics. 2014;30(21):3123–3124. 25061070 10.1093/bioinformatics/btu494 PMC4609014

[26] Liu J, Wu J, Qi F, et al. Natural helper cells contribute to pulmonary eosinophilia by producing IL-13 via IL-33/ST2 pathway in a murine model of respiratory syncytial virus infection. Int Immunopharmacol. 2015;28(1):337–343. 26044350 10.1016/j.intimp.2015.05.035

[27] Em C, Meyerholz DK, Varga SM. IL-13 is required for eosinophil entry into the lung during respiratory syncytial virus vaccine-enhanced disease. J Immunol. 2008;180(4):2376–2384. 18250447 10.4049/jimmunol.180.4.2376

[28] Schwarze J, Cieslewicz G, Hamelmann E, et al. IL-5 and eosinophils are essential for the development of airway hyperresponsiveness following acute respiratory syncytial virus infection. J Immunol. 1999;162(5):2997–3004. 10072551

[29] Qin L, Qiu KZ, Hu CP, et al. Bronchial epithelial cells promote the differentiation of Th2 lymphocytes in airway microenvironment through Jagged/Notch-1 signaling after RSV infection. Int Arch Allergy Immunol. 2019;179(1):43–52. 30943513 10.1159/000495581

[30] Wang J, Chen WD, Wang YD. The relationship between gut microbiota and inflammatory diseases: the role of macrophages. Front Microbiol. 2020;11:1065. 32582063 10.3389/fmicb.2020.01065 PMC7296120

[31] Negi S, Das DK, Pahari S, et al. Potential role of gut microbiota in induction and regulation of innate immune memory. Front Immunol. 2019;10:2441. 31749793 10.3389/fimmu.2019.02441 PMC6842962

[32] Fonseca W, Malinczak CA, Schuler CF, et al. Uric acid pathway activation during respiratory virus infection promotes Th2 immune response via innate cytokine production and ILC2 accumulation. Mucosal Immunol. 2020;13(4):691–701. 32047272 10.1038/s41385-020-0264-z PMC7316593

[33] Wang D, Bai S, Cui Y, et al. Respiratory syncytial virus prevents the subsequent development of ovalbumin-induced allergic responses by inhibiting ILC2 via the IL-33/ST2 pathway. Immunotherapy. 2018;10(12):1065–1076. 30027786 10.2217/imt-2018-0059

[34] Pk P, Harker J, Wang B, et al. Alveolar macrophages are a major determinant of early responses to viral lung infection but do not influence subsequent disease development. J Virol. 2008;82(9):4441–4448. 18287232 10.1128/JVI.02541-07 PMC2293049

[35] MX B, Olsan EE, Rivera-Chávez F, et al. Microbiota-activated PPAR-γ signaling inhibits dysbiotic Enterobacteriaceae expansion. Science (New York, NY). 2017;357(6351):570–575. 10.1126/science.aam9949 PMC5642957 28798125

[36] Atarashi K, Tanoue T, Oshima K, et al. Treg induction by a rationally selected mixture of Clostridia strains from the human microbiota. Nature. 2013;500(7461):232–236. 23842501 10.1038/nature12331

[37] Hayashi A, Sato T, Kamada N, et al. A single strain of Clostridium butyricum induces intestinal IL-10-producing macrophages to suppress acute experimental colitis in mice. Cell Host Microbe. 2013;13(6):711–722. 23768495 10.1016/j.chom.2013.05.013

[38] Cassir N, Benamar S, La Scola B. Clostridium butyricum: from beneficial to a new emerging pathogen. Clin Microbiol Infect. 2016;22(1):37–45. 26493849 10.1016/j.cmi.2015.10.014

[39] Qi F, Bai S, Wang D, et al. Macrophages produce IL-33 by activating MAPK signaling pathway during RSV infection. Mol Immunol. 2017;87:284–292. 28531812 10.1016/j.molimm.2017.05.008

[40] Farlik M, Reutterer B, Schindler C, et al. Nonconventional initiation complex assembly by STAT and NF-kappaB transcription factors regulates nitric oxide synthase expression. Immunity. 2010;33(1):25–34. 20637660 10.1016/j.immuni.2010.07.001 PMC2914224

[41] Ka S, Pletneva LM, Puche AC, et al. Control of RSV-induced lung injury by alternatively activated macrophages is IL-4R alpha-, TLR4-, and IFN-beta-dependent. Mucosal Immunol. 2010;3(3):291–300. 20404812 10.1038/mi.2010.6 PMC2875872

[42] YC L, Zou XB, Chai YF, et al. Macrophage polarization in inflammatory diseases. Int J Biol Sci. 2014;10(5):520–529. 24910531 10.7150/ijbs.8879 PMC4046879

[43] Fo M, Helming L, Gordon S. Alternative activation of macrophages: an immunologic functional perspective. Annu Rev Immunol. 2009;27(1):451–483. 19105661 10.1146/annurev.immunol.021908.132532

[44] Tr M, Coffman RL. TH1 and TH2 cells: different patterns of lymphokine secretion lead to different functional properties. Annu Rev Immunol. 1989;7(1):145–173. 2523712 10.1146/annurev.iy.07.040189.001045

[45] Sm S, Chandrabos C, Yakob E, et al. Memory-T-cell-derived interferon-γ instructs potent innate cell activation for protective immunity. Immunity. 2014;40(6):974–988. 24931122 10.1016/j.immuni.2014.05.005 PMC4105986

[46] Mantovani A, Sica A, Sozzani S, et al. The chemokine system in diverse forms of macrophage activation and polarization. Trends Immunol. 2004;25(12):677–686. 15530839 10.1016/j.it.2004.09.015

[47] Svensson-Arvelund J, Ernerudh J. The role of macrophages in promoting and maintaining homeostasis at the fetal-maternal interface. Am J Reproduct Immunol. 2015;74(2):100–109. 10.1111/aji.12357 25582625

[48] Ge L, Pitman H, Morgan HL, et al. Decidual macrophages: key regulators of vascular remodeling in human pregnancy. J Leukoc Biol. 2016;100(2):315–325. 26819320 10.1189/jlb.1A0815-351R

[49] Hashimoto K, Graham BS, Ho SB, et al. Respiratory syncytial virus in allergic lung inflammation increases Muc5ac and gob-5. Am J Respir Crit Care Med. 2004;170(3):306–312. 15130904 10.1164/rccm.200301-030OC

[50] Mukherjee S, Lindell DM, Berlin AA, et al. IL-17-induced pulmonary pathogenesis during respiratory viral infection and exacerbation of allergic disease. Am J Pathol. 2011;179(1):248–258. 21703407 10.1016/j.ajpath.2011.03.003 PMC3123803

[51] Xm L, Chen X, Gu W, et al. Impaired TNF/TNFR2 signaling enhances Th2 and Th17 polarization and aggravates allergic airway inflammation. Am J Physiol Lung Cell Mol Physiol. 2017;313(3):L592–l601. 28619762 10.1152/ajplung.00409.2016

[52] Sj G, Bb M. IL-17 in the lung: the good, the bad, and the ugly. Am J Physiol Lung Cell Mol Physiol. 2018;314(1):L6–l16. 28860146 10.1152/ajplung.00344.2017 PMC6048455

[53] Rutstein DD, Stebbins RB, Cathcart RT, et al. The absorption and excretion of streptomycin in human chronic typhoid carriers. J Clin Invest. 1945;24(6):898–909. 10.1172/JCI101677 PMC435529 16695287

[54] Sk M, Liu CH, Tzianabos AO, et al. An immunomodulatory molecule of symbiotic bacteria directs maturation of the host immune system. Cell. 2005;122(1):107–118. 16009137 10.1016/j.cell.2005.05.007

[55] Wu S, Rhee KJ, Albesiano E, et al. A human colonic commensal promotes colon tumorigenesis via activation of T helper type 17 T cell responses. Nat Med. 2009;15(9):1016–1022. 19701202 10.1038/nm.2015 PMC3034219

[56] Ii I, Atarashi K, Manel N, et al. Induction of intestinal Th17 cells by segmented filamentous bacteria. Cell. 2009;139(3):485–498. 19836068 10.1016/j.cell.2009.09.033 PMC2796826

[57] Atarashi K, Tanoue T, Shima T, et al. Induction of colonic regulatory T cells by indigenous Clostridium species. Science (New York, NY). 2011;331(6015):337–341. 10.1126/science.1198469 PMC3969237 21205640

[58] JL R, Mazmanian SK. Inducible Foxp3+ regulatory T-cell development by a commensal bacterium of the intestinal microbiota. Proc Natl Acad Sci U S A. 2010;107(27):12204–12209. 20566854 10.1073/pnas.0909122107 PMC2901479

[59] Zhang J, Su H, Li Q, et al. Oral administration of Clostridium butyricum CGMCC0313-1 inhibits β-lactoglobulin-induced intestinal anaphylaxis in a mouse model of food allergy. Gut Pathog. 2017;9(1):11. 28250847 10.1186/s13099-017-0160-6 PMC5322677

[60] Huang H, Liu JQ, Yu Y, et al. Regulation of TWIK-related potassium channel-1 (Trek1) restitutes intestinal epithelial barrier function. Cell Mol Immunol. 2016;13(1):110–118. 25683610 10.1038/cmi.2014.137 PMC4711681

[61] Juan Z, Zhao-Ling S, Ming-Hua Z, et al. Oral administration of Clostridium butyricum CGMCC0313-1 reduces ovalbumin-induced allergic airway inflammation in mice. Respirology. 2017;22(5):898–904. 28122397 10.1111/resp.12985

[62] Bach KKE, Lærke HN, Hedemann MS, et al. Impact of diet-modulated butyrate production on intestinal barrier function and inflammation. Nutrients. 2018;10(10):1499. 10.3390/nu10101499 PMC6213552 30322146

[63] Wang Z, Zhang X, Zhu L, et al. Inulin alleviates inflammation of alcoholic liver disease via SCFAs-inducing suppression of M1 and facilitation of M2 macrophages in mice. Int Immunopharmacol. 2020;78:106062. 31830621 10.1016/j.intimp.2019.106062

[64] Chen G, Ran X, Li B, et al. Sodium butyrate inhibits inflammation and maintains epithelium barrier integrity in a TNBS-induced inflammatory bowel disease mice model. EBioMedicine. 2018;30:317–325. 29627390 10.1016/j.ebiom.2018.03.030 PMC5952406

[65] CJ L, Alley EW, Raval P, et al. Macrophage nitric oxide synthase gene: two upstream regions mediate induction by interferon gamma and lipopolysaccharide. Proc Natl Acad Sci U S A. 1993;90(20):9730–9734. 7692452 10.1073/pnas.90.20.9730 PMC47644

[66] Chang F, Steelman LS, Lee JT, et al. Signal transduction mediated by the Ras/Raf/MEK/ERK pathway from cytokine receptors to transcription factors: potential targeting for therapeutic intervention. Leukemia. 2003;17(7):1263–1293. 12835716 10.1038/sj.leu.2402945

[67] JS P, Lee EJ, Lee JC, et al. Anti-inflammatory effects of short chain fatty acids in IFN-gamma-stimulated RAW 264.7 murine macrophage cells: involvement of NF-kappaB and ERK signaling pathways. Int Immunopharmacol. 2007;7(1):70–77. 17161819 10.1016/j.intimp.2006.08.015

[68] JW P, Kim HY, Kim MG, et al. Short-chain fatty acids inhibit staphylococcal lipoprotein-induced nitric oxide production in murine macrophages. Immune Netw. 2019;19(2):e9. 31089436 10.4110/in.2019.19.e9 PMC6494764

