# Supplementary material for: Oral administration of Clostridium butyricum rescues streptomycin-exacerbated respiratory syncytial virus-induced lung inflammation in mice
Source: Virulence. 2021 Aug 12;12(1):2133–48. doi: 10.1080/21505594.2021.1962137 (PMC8366546; doi:10.1080/21505594.2021.1962137)
Supplement: Supplemental Material [file KVIR_A_1962137_SM9707.zip › Supplementary Table 1.docx]

**Supplementary Table1. Primer sequences used for real time PCR**

|  | Froward primer (5’ - 3’) | Reverse primer (5’ - 3’) |
| --- | --- | --- |
| β-actin | CAACGAGCGGTTCCGATG | GCCACAGGATTCCATACCCA |
| IL-4 | TGTACCAGGAGCCATATCCA | TTCTTCGTTGCTGTGAGGAC |
| IL-5 | GGCTTCCTGTCCCTACTCAT | TCCTCGCCACACTTCTCTTT |
| IL-13 | AGCATGGTATGGAGTGTGGA | TTGCAATTGGAGATGTTGGT |
| IL-10 | GCTCTTGCACTACCAAAGCC | CTGCTGATCCTCATGCCAGT |
| IL-17A | TTTAACTCCCTTGGCGCAAAA | CTTTCCCTCCGCATTGACAC |
| IL-1β | ATCTCGCAGCAGCACATCA | CCAGCAGGTTATCATCATCATCC |
| IFN-γ | TATCTGGAGGAACTGGCAAA | GGTGTGATTCAATGACGCTT |
| Arg1 | AGCTCTGGGAATCTGCATGG | ATGTACACGATGTCTTTGGCAGATA |
| iNOS | CAAGCTGAACTTGAGCGAGGA | TTTACTCAGTGCCAGAAGCTGGA |
| RSV-N | ACCAGAGGTGGCAGTAGAGT | TCACTTGCCCTGCACCATAG |
